# Patient-guided dose reduction of tyrosine kinase inhibitors in chronic myeloid leukaemia (RODEO study): study protocol for a prospective, multicentre, single-arm trial

**DOI:** 10.1186/s12885-023-10697-6

**Published:** 2023-03-10

**Authors:** Melissa F Djodikromo, Rosella PMG Hermens, Bart JF van den Bemt, Yolba Smit, Tim M Govers, Charlotte L Bekker, Nicole MA Blijlevens

**Affiliations:** 1 Department of Haematology, Radboud university medical center, Nijmegen, The Netherlands; 2 Scientific Institute for Quality of Healthcare (IQ healthcare), Radboud university medical center Nijmegen, Nijmegen, The Netherlands; 3 Department of Pharmacy, Sint Maartenskliniek, Nijmegen, The Netherlands; 4 Department of medical imaging, Radboud university medical center, Nijmegen, The Netherlands; 5 Department of Pharmacy, Radboud university medical center, Nijmegen, The Netherlands

**Keywords:** Chronic myeloid leukaemia, Tyrosine kinase inhibitors, Dose reduction, Shared decision making, Patient-centred care

## Abstract

**Background:**

Dose reduction of tyrosine kinase inhibitors (TKI) in patients with chronic myeloid leukaemia (CML) with an optimal response to TKIs may support cost-effective medication use by maintaining therapeutic effectiveness while reducing adverse events and medication costs. As the choice for dose reduction depends on patients’ individual needs and preferences, a patient-centred approach is warranted. Therefore, a study to evaluate the effectiveness of patient-guided dose reduction in patients with CML who are in a major or deep molecular response is designed.

**Methods:**

This study is a prospective, multicentre, single-arm study. 147 patients with CML (aged ≥ 18 years) in chronic phase, who are treated with imatinib, bosutinib, dasatinib, nilotinib or ponatinib, and have reached at least major molecular response (defined as having BCR-ABL levels < 0.1% for an uninterrupted period of 6 months) are eligible. Patients will use an online patient decision aid and a shared decision making consultation will be held, after which patients who choose to will receive a personalised, lower TKI dose. Primary outcome is the proportion of patients with intervention failure at 12 months after dose reduction, defined as patients who have restarted their initial dose due to (expected) loss of major molecular response. For this, BCR-ABL1 levels will be analysed from blood samples drawn at baseline, 6 weeks after dose reduction and 3-monthly thereafter. Secondary outcomes include the proportion of patients with intervention failure at 6 and 18 months after dose reduction. Other outcomes include differences before and after dose reduction regarding the number and severity of patient-reported side effects; quality of life; beliefs about medicines; and medication adherence. Patients’ level of decisional conflict and regret after choosing dose reduction will be assessed, as will the decisional process experienced by patients and healthcare providers.

**Discussion:**

Outcomes of this trial using a personalised approach will provide clinical and patient-reported data to guide future dose reduction of TKIs in CML. If the strategy appears to be effective, it may be implemented as another valid option to offer next to standard of care to prevent potential unnecessary exposure to higher TKI doses in this selected group of patients.

**Trial registration:**

EudraCT number 2021-006581-20.

**Supplementary Information:**

The online version contains supplementary material available at 10.1186/s12885-023-10697-6.

## Background

Chronic myeloid leukaemia (CML) is a rare haematologic malignancy, with a crude annual incidence of 0.7-1.0/100,000 in the Netherlands [1]. Treatment of chronic phase CML often consists of lifelong treatment with a tyrosine kinase inhibitor (TKI): imatinib, bosutinib, dasatinib, nilotinib or ponatinib [2]. Targeting the BCR-ABL1 oncogene, these TKIs have resulted in significantly improved prognosis, leading to a near to normal life expectancy if adequately treated [3–5]. Despite its effectiveness, CML treatment implicates lifelong treatment for the majority of patients whereas half of the patients experience mild (e.g. fatigue and nausea), to more severe side effects (e.g. pleural effusion, cardiovascular events, diarrhoea and hepatotoxicity) [2, 6–9]. These side effects decrease patients’ quality of life, negatively influence adherence, make patients switch medication, and cause significant morbidity and mortality for some patients [10, 11]. As such, interventions to reduce the TKI-related side effects while maintaining molecular response (i.e. stable disease) are needed.

Dose reductions of TKIs in patients with CML are often for the management of side effects and is recommended by existing CML treatment guidelines and the summary of product characteristics of the TKIs [2, 12–21]. Additionally, dose reductions in patients with CML have been studied (1) in newly diagnosed patients with CML as frontline TKI therapy, (2) to prevent future toxicity and (3) in patients with at least stable MMR; either 3a) as continuous maintenance therapy or 3b) prior to a treatment discontinuation attempt, indicating that dose reductions may be applied throughout the patients’ treatment journey [22–31]. These studies demonstrated that the majority of patients with CML in MMR and DMR can successfully reduce their TKI dose without hampering the therapeutic effectiveness [22–31]. Although dose reduction seems potentially effective, many issues remain unsolved, including dose and schedule for each individual, as well as the identification of the best dose reduction strategy to reduce the frequency and severity of side effects.

A major limitation of these studies is their one-size fits all dose-reduction approach for study participants. Moreover, dose reduction was initiated by the healthcare provider, with no patient involvement. However, considering patients’ concerns and preferences regarding dose reduction is essential to increase willingness to receive a lower dose and promote treatment adherence. Also, studies suggest that haematologic patients wish to be involved in treatment decision making, and prefer shared decision making [32–34]. Nonetheless, no study has explored patient-guided dose reduction and the effect of it on molecular responses. The aim of the RODEO study is therefore to assess the effectiveness of patient-guided dose reduction in patients with CML who are in stable MMR or DMR. This paper describes the design of the RODEO study.

## Objectives

### Primary objective

The primary objective of this study is to assess the proportion of patients with intervention failure at 12 months after dose reduction, defined as patients who have restarted their initial dose due to (expected) loss of MMR.

### Secondary objectives

- To assess the proportion of patients with intervention failure at 12 months.
- To assess the proportion of patients with intervention failure at:
  - 6 months after dose reduction.
  - 18 months after dose reduction.
- To compare the following patient-reported outcomes before dose reduction and 12 months after dose reduction:
  - the proportion of patients with (patient-reported) side effects, including number of side effects (per patient and total) and severity of side effects.
  - Patients’ quality of life.
  - Patients’ beliefs about medicines.
  - Medication adherence.
  - Healthcare consumption and productivity loss.
- To assess patient involvement during shared decision making from the perspectives of patients and healthcare providers.
- To assess patients’ decisional conflict and regret after dose reduction.
- To explore patient and treatment factors influencing successful dose reduction (e.g. level and duration of MMR before reduction, duration and type of TKI treatment).

## Methods

This manuscript was written in accordance with the Standard Protocol Items: Recommendations for Interventional Trials (SPIRIT) guidelines [35]. A completed SPIRIT checklist is presented in [Additional file 1].

### Study design

**Fig. 1:**
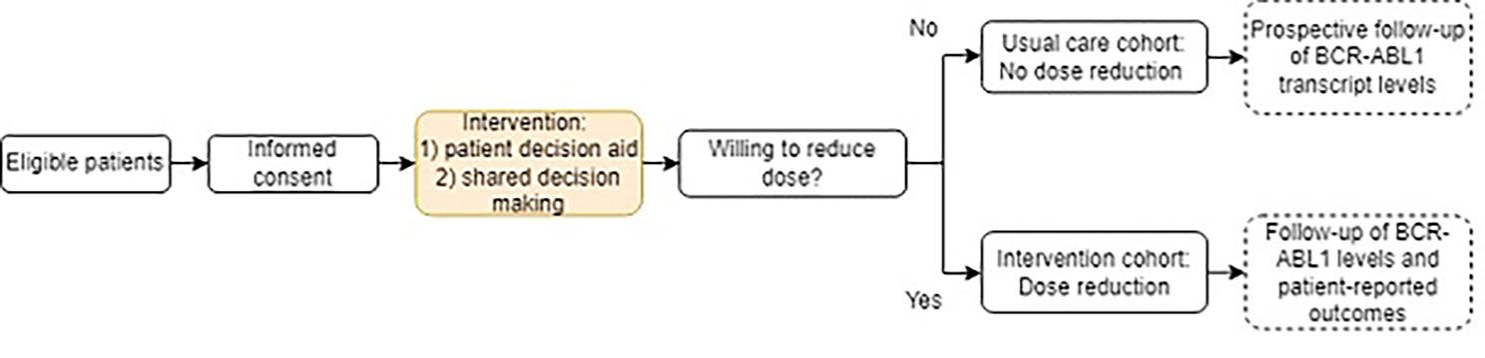
Overall Study Scheme

The RODEO study is a prospective, multicentre, single arm study evaluating the effectiveness of patient-guided dose reduction of TKIs in optimally responding patients with CML. The study is being conducted at ten Dutch hospitals, including academic as well as peripheral hospitals. A study flowchart is presented in [Fig. 1].

### Participants

The study population will include adult patients, aged ≥ 18 years, diagnosed with chronic phase CML, using a TKI (imatinib, bosutinib, dasatinib, nilotinib, ponatinib) who have reached optimal treatment response i.e. at least MMR. A previous participation in a clinical trial does not represent a reason for exclusion from the present study.

#### Inclusion criteria

In order to be eligible to participate in this study, a subject must meet all of the following criteria:

- Aged ≥ 18 years.
- Diagnosed with chronic phase CML.
- treated with a TKI (imatinib, bosutinib, dasatinib, nilotinib, ponatinib, there are no restrictions regarding using a lower than standard dose at inclusion, or previously having switched from TKI due to toxicity.
- MMR or DMR for an uninterrupted period of at least 6 months at inclusion date.
- Able and willing to participate.
- Has provided written informed consent.

#### Exclusion criteria

A (potential) subject who meets any of the following criteria will be excluded from participation in this study:

- Inability to understand the nature and extent of the trial and the procedures required (left at the discretion of the treating physician).
- Previous loss of MMR on a reduced TKI dose due to intolerability.
- Molecular or cytogenetic failure to previous TKI.
- Previous allogeneic hematopoietic stem cell transplantation.
- CML in accelerated phase or blast crisis.
- Pregnancy or lactation.
- Life expectancy ≤ 1 year.

### Patient recruitment

The haematologist or (hemato-)oncology nurse will screen patients on eligibility for participation on in- and exclusion criteria. During a regular consult, eligible patients are verbally asked to participate. Written informed consent will be obtained subsequently. Consenting patients will receive the intervention, as described below.

### Intervention: patient-guided dose reduction

#### Dose reduction

**Fig. 2:**
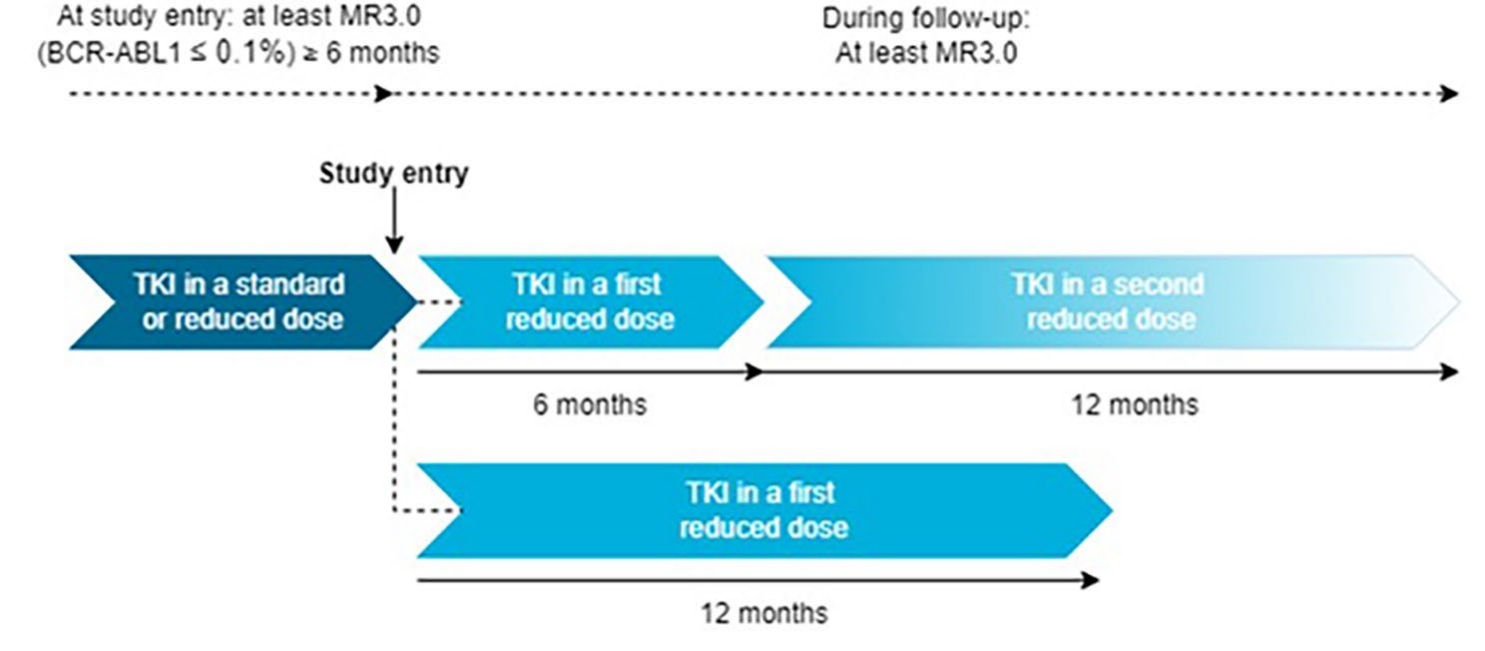
Patients can opt for two dose reductions, leading to a total follow-up of 18 months

Participants will receive a lower TKI dose that is individualised using a patient decision aid and decided upon during a shared decision making consultation. If wanted and still in MMR, patients can reduce their TKI dose again at six months after the first dose reduction [Fig. 2]. Dose reduction is maximised by 50%, depending on the patient’s baseline molecular status and available dosages for each TKI, as shown in Table 1. Patients will remain on the lower TKI dose until intervention failure or until the patient wishes to restart their initial dose.

**Table 1:** Quantitative dose reduction steps according to baseline molecular status

Baseline molecular status	Maximum dose reduction 1 (compared to patient’s starting dose)	Maximum dose reduction 2 (compared to reduced dose)
MR3-MR4 (BCR-ABL1 levels ≤ 0.1% and 0.01% on the International Scale (IS), respectively)	25%	25%
MR4.5-MR5 (BCR-ABL1 levels ≤ 0.0032% and 0.001% on the IS, respectively)	25%	50%

#### Patient decision aid

A Dutch patient decision aid (PtDA), www.keuzehulpcml.nl, will be used to support patients in the decision to lower their TKI dose. Consenting patients will receive the PtDA from the local study team. The PtDA was developed following an iterative and systemic process as described by Coulter et al., the guideline for DAs from the Dutch Patient Federation and in accordance with the international patient decision aid standards (IPDAS) criteria [41–43]. The PtDA offers several steps through which the patient can navigate: It explains the treatment options (dose reduction vs. no dose reduction), as well as a brief overview of the differences and similarities between the two options. The PtDA includes a number of questions to test patients’ knowledge about the treatment options, as well as questions to help CML patients clarify their values.

#### Shared decision making

During a following shared decision making consultation, the results of the PtDA will be reviewed, patients’ willingness to lower their TKI dose will be discussed as well as to which extent the patient is willing to lower their dose using shared decision making. A tailored dose reduction will be defined subsequently. To achieve shared decision making, haematologists and nurses will be trained on shared decision making using an e-learning.

### Study outcomes

#### Primary outcome

The primary study outcome is the proportion of patients with intervention failure at 12 months after first dose reduction.

#### Definition of intervention failure

Intervention failure is defined as restart of the initial dose due to (expected) loss of MMR, a decision left at the discretion of the treating haematologist.

Loss of MMR is confirmed if any single sample detects ≥ 0.1% BCR-ABL1 transcript levels (IS). If loss of MMR occurs, patients are required to restart the TKI dose prior to study entry as soon as possible. The patient will be followed until MMR is regained. The treating haematologist will decide on further procedures.

Expected loss of MMR, e.g. due to ≥ 1 log increase compared to baseline molecular status, is left at the discretion of the treating haematologist. In case of doubt, consultation of the main investigator of the study team is advised. In case of restart of the initial dose, the reason, date and molecular status at that time point will be registered in the eCRF and the treating haematologist will decide on further procedures.

For this, the most recent BCR-ABL1 level (no older than 6 weeks) will be used as baseline BCR-ABL1 measurement. Six weeks after dose reduction 1 and 2, an extra blood sample is drawn. Thereafter, monitoring of BCR-ABL1 transcript levels will be performed 12-weekly following monitoring recommendations by the European LeukemiaNet [2]. All BCR-ABL results are seen and authorised by the internist-haematologist. An increase in the BCR-ABL1 transcript levels and possible loss of response is therefore noticed in a sufficient early stage upon which the treating haematologist can act. Literature show that in case of relapse, resuming standard dose allows for MMR reachievement within 4 months, whereas no haematological relapses have been described [26, 27, 31].

#### Secondary outcomes

Changes before and after dose reduction will be assessed in terms of the proportion of patients with (patient-reported) side effects, including number of side effects (per patient and total) and severity of side effects, patients’ quality of life, beliefs about medicines, medication adherence, healthcare consumption and productivity loss. Furthermore, patients’ level of decisional conflict and regret after dose reduction and patient involvement during shared decision making will be evaluated. All secondary study outcomes will be assessed at pre-defined time points using several questionnaires, as described in the following sections and shown in [Supplementary Tables 2, Additional file 2].

#### Patient-reported side effects

The EORTC QLQC30-CML24 is a validated questionnaire that assesses health related quality of life, including CML specific symptoms [36]. Items in the questionnaire include questions about symptom burden ), impact on daily life and worry/mood, body image problems, and satisfaction with care, information and social life. This will be combined with the EORTC Symptom list that focusses on 17 additional symptoms in-depth [37]. (patient-reported) Side effects will be assessed at baseline, at 6 weeks after dose reduction, month 3 and then 3-monthly until study end.

#### Health related quality of life

Patients’ quality of life will be measured using the EORTC QLQC30-CML24 (described above) and the EQ-5D-5 L questionnaire. The EQ-5D-5 L is a standardised measure of health status and includes statements about mobility, self-care, activities, pain/discomfort and anxiety/depression [38]. Also, patients are asked to score their health on a scale from 0 to 100. The scores on the EQ-5D-5 L questionnaire reflect 5 levels of perceived problems related to health status (i.e., level 1 indicates no problem, whereas level 5 indicates extreme problems). Quality of Life will be assessed at baseline, month 6 and month 12 of follow-up.

#### Beliefs about medicines

Patients’ beliefs about medicines will be evaluated using the Beliefs about Medicine Questionnaire Specific (BMQ-S) to assess patients’ views on their own specific medicines [39]. All items are rated on a 5-point Likert scale. The BMQ-specific consists of 5 statements about the necessity of the prescribed medication and 5 statements concern possible negative side effects / consequences. The BMQ-S will be assessed at baseline, month 6 and month 12 of follow-up.

#### Medication adherence

The Medication Adherence Report Scale (MARS-5© Prof Rob Horne) consists of 5 statements concerning forgetting, dose changing, stopping, skipping and using less medication than prescribed [40]. Participants indicate the frequency on a 5-point scale (always – often – sometimes – rarely – never) for each question, with ascending scores from always (1 point) to never (5 points). Scores are summed up, ranging from 5 to 25. At a sum score of > 21 or a score 4 on each individual item, the patient is considered adherent. Medication adherence will be measured at baseline and at 6- and 12-months follow-up.

#### Health consumption and work productivity loss

Healthcare resource use and productivity will be collected using the Medical Consumption questionnaire (iMCQ) and the Productivity Cost Questionnaire (iPCQ)[41, 42]. The iMCQ includes questions about whether patients have visited or consulted healthcare providers. The iPCQ maps the costs of reduced productivity in paid and unpaid work. Both questionnaires will be assessed at baseline, and at 6- and 12-months follow-up.

### Process evaluation outcome measures

#### Patient involvement during shared decision making

The first consult of each involved haematologist with the first involved study patient will be audio-recorded and rated with the Observer OPTION (Observing patient involvement) 5- item by an external party. The Observer OPTION consists of a set of competences, including problem definition, explaining legitimate choices, portraying options and communication risk, and conducting the decision process. The instrument aims to measure to what extent the patient is involved in the decision about the treatment and consists of 5 items [43, 44]. Healthcare providers will be given the opportunity to receive verbal and written feedback after their first shared decision making consult.

Furthermore, the SDM-Q9 (for patients) and SDM-Q-DOC (for healthcare providers) questionnaires will be used to assess the process of SDM from the perceptions of patients and healthcare providers, respectively. Both instruments consist of nine statements, which can be rated on a six-point scale from 0 (completely disagree/not applicable at all) to 5 (completely agree/completely applicable). Summing all items leads to a raw total score between 0 and 45 [45]. The SDM-Q9 will be assessed once directly after the shared decision making consult, whereas the SDM-Q-doc will be assessed after the third SDM consult of each participating healthcare provider.

#### Patients’ level of decisional conflict and regret

The effect of the decision to lower TKI dose on the level of distress will be evaluated among patients at 6 weeks follow-up using the Decisional conflict scale (DCS), a 16-item questionnaire for assessment of uncertainty in choosing options, factors contributing to uncertainty, and effective decision making. At 12-months follow up we will measure regret or remorse using the Decisional regret scale (DRS) [46, 47].

### Asessments

#### Baseline assessment

Baseline status will be recorded after patients decide to reduce their TKI dose and within 6 weeks of actually starting their reduced dose. Baseline data contain the following information:

1. Data from the patient’s electronic medical file:

- Patient characteristics: gender, age, educational level, comorbidities.
- Treatment characteristics: date of diagnosis, treatment duration, name and dose of (previously received) TKI.
- Response history: Duration and level of molecular response (MR3 – MR6).
- Baseline BCR-ABL1 transcript level: The most recent BCR-ABL1 level (not older than 6 weeks) will be used as baseline BCR-ABL1 measurement.

2) Patient-reported data.

- (patient-reported) side effects (EORTC QLQ30-CML24).
- Quality of Life (EQ-5D-5 L).
- Beliefs about Medicine (BMQ).
- Healthcare consumption (iMCQ/iPCQ).
- Medication adherence (MARS-5).

#### Follow-up assessments

Follow-up data consist of BCR-ABL1 transcript levels 6 weeks after dose reduction 1 and 2. Thereafter, monitoring will be performed 12-weekly following monitoring recommendations by the European LeukemiaNet [2] until the follow-up period is completed. Optionally, extra blood will be drawn to study relevant research questions that arise during this study. Patient-reported data as defined in Secondary outcomes will be collected at pre-defined timepoints as summarised in [Supplementary Tables 2, Additional file 2].

### Data collection and management

Data will be collected on electronic Case Report Forms (eCRF) to document eligibility, effectiveness parameters, and parameters necessary to evaluate the study endpoints. A unique patient Record ID will be assigned to each enrolled patient by the electronic Case Report File (eCRF) during the registration. Data consists of patient blood samples and questionnaires. Blood samples may be stored at the study site and used for additional scientific research in the context of dose reduction for a maximal period of 20 years if permission hereof was documented in the patient informed consent form, otherwise blood samples will be disposed. All questionnaires will be sent automatically via Castor Electronic Data Captur (Castor EDC) to participants by email. An automatic reminder will be sent after 7 days. Data concerning participants will be kept confidential and will only be accessible for study team members. Data will be coded and kept based on the rules for good clinical practice (GCP) and according to the data management plan. Personal data will be handled in accordance with the General Data Protection Regulation (AVG).

### Sample size calculation

The primary objective of this study is to assess the proportion of patients with treatment failure at 12 months after dose reduction, defined as patients who restart their initial dose due to (expected) loss of MMR.

The tested hypothesis will be:

H0: Proportion of patients with treatment failure at 12 months after dose reduction > 28%

H1: Proportion of patients with treatment failure ≤ 28%

A previous study of Clark et al. showed that 19% (90% CI 9.5–28.0%) of patients on reduced TKI dose lost MMR, of which all regained MMR within 4 months after restarting their initial dose [26, 27]. Intervention failure in 19% of patients is considered clinically acceptable by expert consultation. For this study, a sample size of n = 140 is warranted to find a two-sided 90% CI (Clopper-Pearson) below the upper limit of 28% with a power of at least 0.80, assuming a probability of 0.19 for treatment failure. Considering an expected dropout of 5%, the sample size is 147 patients of patients who choose to reduce their TKI dose.

### Data analysis

All statistical analyses will be performed using IBM SPSS Statistics version 25.0. All individual data will be listed. Adherence to the protocol (e.g. inclusion – and exclusion criteria, times of measurement, completeness and consistency of data) will be checked using the recorded data and descriptively presented. Descriptive variables will be provided using mean +/−SD, median (p25-p75) or frequencies/percentages as appropriate. Missing data on determinants/covariates will be described using descriptive analyses. A more detailed statistical analysis plan (SAP) providing all details on the statistical evaluation will be prepared before database lock.

#### Primary endpoint

The primary endpoint is the proportion of patients who experience intervention failure, defined as patients who have restarted their initial dose due to (expected) loss of MMR.

#### Interim analysis

With a futility interim analysis, we aim to obtain a first insight on the feasibility of dose reduction in clinical practice. This analysis will be performed when half of the patients (n = 70) are included and followed for a duration of 6 months. The proportion of patients who experienced treatment failure will be calculated as follows: *[number of patients with treatment failure / number of recruited patients].*

If at the interim analysis the proportion of patients lost MMR is considered too high as judged by the study team, the study will be suspended. If 75% of the patients have restarted their initial dose due to (expected) loss of MMR at the time of the interim analysis, the study will be suspended. Testing of the primary endpoint will only be performed at the end of the study.

#### Final analysis

The final analysis will be performed after all 140 patients are at the end of their study period. If the upper limit of the two-sided exact 90% CI (Clopper-Pearson) is < 28%, the study will be considered successful. This is equivalent to a one-sided test at alpha = 0.05. Apart from analyses for all patients, stratified analyses for the different TKIs and molecular response (MMR, MR4, MR4.5, MR5, MR6 including duration) will be performed.

#### Secondary endpoints

All questionnaires will be analysed using their validated protocols. We will descriptively report percentages or means over the three timepoints (i.e. baseline, 6 months after dose reduction and 12 months after dose reduction), together with their 95% CI, as appropriate.

Patient involvement during shared decision making as well as patients’ level of decisional conflict and regret after dose reduction will only be assessed once after dose reduction. Means, together with their 95% CI will be descriptively presented.

## Discussion

Dose reduction of TKIs among patients with CML who have an optimal response to TKIs has been evaluated in few prospective clinical trials or assessed retrospectively using real-world data. However, the one-size fits all approach of studies and the lack of patient involvement in treatment decision making do not respond to a personalised approach and highlight the need for the prospective RODEO study.

Shared decision making (SDM), a process which supports decision making in preference-sensitive decisions like dose reduction, fits well with this need. SDM has shown to increase patients’ satisfaction with a decision and is an important factor for compliance with and adherence to cancer treatment [48]. The use of patient decision aids has become an increasingly popular approach to help patients take an active role in the decisional process and hereby facilitate shared decision making [49]. By combining the use of a patient decision aid, training healthcare providers in SDM and providing SDM consults, the RODEO study responds to patients’ needs and preferences. This unique, personalised approach to dose reduction is not only ethically warranted, but will also foster successful and sustainable implementation of dose reduction strategies. It can be expected that when patients’ concerns and preferences regarding dose reduction are taken into account, the likelihood of successful dose reduction is increased. By combining clinical, patient-reported and costs outcomes, the RODEO study is designed to evaluate the effectiveness and costs of a patient-guided dose reduction strategy.

Although ideally, a randomised controlled trial (RCT) design would be the preferred design of testing the effectiveness of an intervention.However, designing a RCT of sufficient power generally requires a large sample size, which is hardly feasible in the Netherlands as CML is a rare disease with a low number of patients. For this reason, a patient-preference trial also was no option and a prospective single-arm trial design with sufficient power was chosen to evaluate our strategy.

A major strength of RODEO study is the variety of outcome measures which are prospectively assessed: This study will not only evaluate clinical outcomes (i.e. molecular responses) to lower TKI doses, but will also assess patient-reported outcomes on side effects, quality of life, beliefs about medicines, medication adherence and healthcare consumption, which could give an insight in the impact of dose reduction from the patient’s perspective. Furthermore, the RODEO study has broad eligibility criteria and will include diverse patients in terms of TKI dosage (a standard, increased or already reduced dose) and line of treatment. This clinical diversity increases external validity – especially ecological validity and the generalisability of its findings. This study protocol also has potential limitations. As this is a single-arm trial, the risk of selection bias arises, as only patients who are supportive of dose reduction might participate. To gain insight into possible selection bias, we will register patient characteristics of non-participants, including their reasons not to participate Also, with no control arm, it remains unknown whether the same results would also have been demonstrated as a result of natural course.

Currently, the possibility of dose reduction is limited to the management of side effects, as described in the SmPCs and (inter)national guidelines [2, 12–21]. However, with this patient-guided dose reduction strategy, personalised dose reduction can be made available for patients with stable disease who have diverse treatment goals, i.e. continuous maintenance therapy, gradual tapering prior to a discontinuation attempt and even after a failed discontinuation attempt. To conclude, more knowledge on patient-guided dose reduction of TKIs may contribute to optimal, patient-centred and cost-effective TKI use. If the strategy appears to be effective, it may be implemented as another valid option to offer next to standard of care to prevent potential unnecessary exposure to higher TKI doses.

## Trial status

The trial started on June 14th 2022 and is currently recruiting. Current protocol version 1.1, date September 7th 2022.

## Electronic supplementary material

Below is the link to the electronic supplementary material.

Supplementary Material 1

Supplementary Material 2

## Data Availability

Not applicable.

## Abbreviations

AVG: General Data Protection Regulation (DutchAlgemene verordening gegevensbescherming)
BMQ-S: Beliefs about Medicine Questionnaire Specific
CML: Chronic myeloid leukaemia
DCS: Decisional conflict scale
DMR: Deep molecular response
DRS: decision regret scale
eCRF: Electronic Case Report Forms
EDC: Electronic Data Capture
EORTC: European Organisation for Research and Treatment of Cancer
GCP: Good clinical practice
iMCQ: Medical Consumption questionnaire
iPCQ: Productivity Cost Questionnaire
IPDAS: International patient decision aid standards
MARS: Medication Adherence Report Scale
METC: Medical Ethical Committee (Dutch: Medisch Etische Toetsings Comissie)
MMR: Major molecular respons
PtDA: Patient decision aid
QLQ-C30: Quality of Life of Cancer Patients
RCT: Randomised controlled trial
SPIRIT: Standard Protocol Items: Recommendations for Interventional Trials
TFR: Treatment-free remission
TKI: Tyrosine kinase inhibitor
