# Supplementary material for: Patient-guided dose reduction of tyrosine kinase inhibitors in chronic myeloid leukaemia (RODEO study): study protocol for a prospective, multicentre, single-arm trial
Source: BMC Cancer. 2023 Mar 10;23:231. doi: 10.1186/s12885-023-10697-6 (PMC10007754; doi:10.1186/s12885-023-10697-6)
Supplement: Supplementary file 2 — Supplementary Material 2 [file 12885_2023_10697_MOESM2_ESM.docx]

Table 2 Schedule of measurements.

| Study period | | | | | | | | | | |
| --- | --- | --- | --- | --- | --- | --- | --- | --- | --- | --- |
|  | Screening & enrolment | Baseline |  | Follow-up | | | | | | |
| Timepoint |  | T_-6w_ | T_0_: DR1 | W_6_ | W_12_ | M_6_  _optional: start DR2*_ | W_6_ _after DR 2*_ | M_9_ | M_12_ | M_18*_ |
| **Enrolment** |  |  |  |  |  |  |  |  |  |  |
| - Eligibility screening | X |  |  |  |  |  |  |  |  |  |
| - Informed consent | X |  |  |  |  |  |  |  |  |  |
| - Medical history | X |  |  |  |  |  |  |  |  |  |
| **Assessments** |  |  |  |  |  |  |  |  |  |  |
| - BCR-ABL |  | X |  | X | X | X | X | X | X | X |
| - (patient-reported) side effects (EORTC QLQ30-CML24) |  | X |  | X | X | X | X | X | X | X |
| - Quality of Life (EQ-5D-5L) |  | X |  |  |  | X |  |  | X |  |
| - Beliefs about Medicine (BMQ) |  | X |  |  |  | X |  |  | X |  |
| - Medication Adherence (MARS-5) |  | X |  |  |  | X |  |  | X |  |
| - Healthcare consumption (iMCQ/iPCQ) |  | X |  |  |  | X |  |  | x |  |
| - Process of SDM (Observer OPTION 5) |  |  | X (during first SDM consult) |  |  |  |  |  |  |  |
| - Process of SDM (SDM-Q9 |  |  | X (directly after SDM consult) |  |  |  |  |  |  |  |
| - Process of SDM (SDM-Q-doc) |  |  | X (after the healthcare provider’s third consult |  |  |  |  |  |  |  |
| - Decisional conflict (DRS, DCS) |  |  |  | X_DCS_ |  |  |  |  | X_DRS_ |  |

*: a second dose reduction at week 24 after the first dose reduction is optional. Two extra measurements (marked in orange) are assessed if patients opt for a second dose reduction.
